# Effects of Aminoglycoside Antibiotics on Human Embryonic Stem Cell Viability during Differentiation In Vitro

**DOI:** 10.1155/2017/2451927

**Published:** 2017-09-24

**Authors:** Divya S. Varghese, Shama Parween, Mustafa T. Ardah, Bright Starling Emerald, Suraiya A. Ansari

**Affiliations:** ^1^Department of Biochemistry, College of Medicine and Health Sciences, UAE University, Al Ain, Abu Dhabi, UAE; ^2^Department of Anatomy, College of Medicine and Health Sciences, UAE University, Al Ain, Abu Dhabi, UAE

## Abstract

Human embryonic stem cells (hESCs) are being used extensively in array of studies to understand different mechanisms such as early human embryogenesis, drug toxicity testing, disease modeling, and cell replacement therapy. The protocols for the directed differentiation of hESCs towards specific cell types often require long-term cell cultures. To avoid bacterial contamination, these protocols include addition of antibiotics such as pen-strep and gentamicin. Although aminoglycosides, streptomycin, and gentamicin have been shown to cause cytotoxicity in various animal models, the effect of these antibiotics on hESCs is not clear. In this study, we found that antibiotics, pen-strep, and gentamicin did not affect hESC cell viability or expression of pluripotency markers. However, during directed differentiation towards neural and hepatic fate, significant cell death was noted through the activation of caspase cascade. Also, the expression of neural progenitor markers Pax6, Emx2, Otx2, and Pou3f2 was significantly reduced suggesting that gentamicin may adversely affect early embryonic neurogenesis whereas no effect was seen on the expression of endoderm or hepatic markers during differentiation. Our results suggest that the use of antibiotics in cell culture media for the maintenance and differentiation of hESCs needs thorough investigation before use to avoid erroneous results.

## 1. Introduction

Antibiotics are routinely used in long-term stem cell cultures in the laboratories to avoid general bacterial contamination. Penicillin-streptomycin (pen-strep) is one of the most commonly used antibiotics in the cell culture media to control bacterial contamination. However, many strains of bacteria are found to be resistant to pen-strep. In these situations, other broad spectrum antibiotics such as normocin and gentamicin are used [1]. Cytotoxic effects of gentamicin have been reported in animal models (for a review, see [2]). Gentamicin is also widely used for the treatment of infections caused by gram-negative bacteria. In animal and human models, the use of gentamicin is reported to cause ototoxicity and nephrotoxicity [3, 4]. Animals treated with high therapeutic doses of gentamicin show extensive necrosis of proximal kidney tubular cells [4] while low doses of gentamicin induced programmed cell death through the activation of caspase cascade [5]. In addition, therapeutic doses of gentamicin have been shown to cause hearing loss and nephrotoxicity in neonates [6, 7]. Although it is known that aminoglycosides can cross placenta, the effect of maternal use of these antibiotics on early embryonic development if any is still not well known.

Human embryonic stem cells (hESCs) are pluripotent cells which can be differentiated into all three germ layers, the ectoderm, mesoderm, and endoderm, and the protocols for the directed differentiation of hESCs towards specific cell lineages have been published [8–11]. The availability of hESC-derived cell lines had opened up the possibility to detect cytotoxicity of various drugs as well as the possibility to use them as a developmental model to understand the effect of different toxins or teratogens on early human embryogenesis which is otherwise possible only in animal models.

Since gentamicin can cross the placenta during pregnancy, it may cause adverse effects on the developing organs of the fetus. This study was therefore designed to understand the effect of routinely used antibiotics such as pen-strep and gentamicin on hESC proliferation and their differentiation towards neural and hepatic fate keeping in mind that, this might also help to understand the side effects of these aminoglycosides in early human embryogenesis in vivo.

## 2. Materials and Methods

### 2.1. Cell Culture, Differentiation, and Antibiotic Treatment

hESCs (H9, WiCell Institute) were maintained in feeder-free condition on Matrigel- (Corning, cat. number 354227) coated plates in mTeSR1 medium (Stem Cell Technologies, cat. number 05850) and were between passages 37 to 46 in all of the experiments. Neural induction protocol was replicated as published previously [11, 12]. Briefly, 50,000 cells/cm^2^ were plated on a 24 well plate coated with Matrigel and maintained in mTeSR1 medium until fully confluent. The medium was then replaced with neural induction medium containing KSR media (15% Knockout Serum Replacement (KO-SR Gibco, cat. number 10828028), 1% L-glutamine (100x-Gibco, cat. number 25030081), 1% MEM (Hyclone, cat. number SH40003.01), and 0.1% beta-mercaptoethanol (Gibco, cat. number 31350010) in knockout DMEM (Gibco, cat. number 10829018) supplemented with LDN193189 (Stem Cell Technologies, cat. number 72142-1 mg lot number SCO4565), inhibitor BMP type 1 receptors (100 nM) and SB431542 (Milipore, cat. number 616461-5 mg Lot number D00165595), and activin receptor inhibitor (10 *μ*M) and N2 media (8.5 mM glucose, 1x N-2 supplement (Gibco, cat. number 17502048) in DMEM/F12 (Gibco, cat. number 17330-032, 1 : 1)). The initial 5 days of differentiation included 100% of KSR media which was gradually replaced with N2 media from day 5 onwards as 25% N2, 50% N2, 75% N2, and 100% N2. The differentiation was carried out for a total of 12 days.

For hepatic differentiation, we adopted protocols published before [10, 13]. Briefly, H9 cells were cultured in feeder-free condition on Matrigel-coated plates in mTeSR1 medium until 70–80% confluent. At this point, differentiation was induced by replacing mTeSR1 media with definitive endoderm (DE) induction media comprised of RPMI-1640 (Gibco, cat. number 11875093) supplemented with 2% B27 (Gibco, cat. number 17504044), 100 ng/ml Activin A (R&D Systems, cat. number 338-AC-050), 50 ng/ml Wnt3a (R&D Systems, cat. number 5036-WN/CF), and 1 mM Na butyrate (Sigma-Aldrich, cat. number 303410) for the first 24 h. The media was changed with the concentration of Na butyrate reduced to 0.5 mM and cultured with media change every 24 h. After 5 days of culture, media was changed to hepatocyte differentiation media containing KO-DMEM supplemented with 20% KO-SR, 0.5% L-glutamine, 1% nonessential amino acids, 0.1 mM *β*-mercaptoethanol, and 1% DMSO (Sigma-Aldrich, cat. number D8418) and cultured for 5 more days to get hepatic progenitor cells. Antibiotics, penicillin-streptomycin (10,000 U/ml, cat. number 15140122), and gentamicin (50 mg/ml, cat. number 15750060) were purchased from Thermo Fisher Scientific and used as indicated in the text.

### 2.2. RNA Isolation and RT-PCR

Total RNA was isolated using MasterPure™ RNA Purification Kit from Epicentre (cat. number MCR 85102) according to the manufacturer's instructions. For cDNA synthesis, 1.0 *μ*g of total RNA was reverse transcribed in a 20 *μ*l reaction using SuperScript® VILO cDNA Synthesis Kit (cat. number 11754050) as per manufacturer's instructions. Real-time qRT-PCR was performed using SYBR® Green Real-Time PCR Master Mix (Thermo Fisher Scientific) in a 10 or 20 *μ*l reaction volume. The primer sequences used in the real-time PCR reactions were obtained from Primer Bank (https://pga.mgh.harvard.edu/primerbank/index.html) as listed in Table 1.

### 2.3. Protein Extraction and Quantitation

Undifferentiated-, neural-, or hepatic-differentiated hESCs were cultured in 24- (Corning, cat. number 3527) or 4-well plates (Thermo Scientific, cat. number 176740), precoated with human ESC qualified Matrigel (Corning, cat. number 354227). The cells were rinsed with sterile 1x-D-PBS (Stem Cell Technologies, cat. number 37350) and were gently scraped using a sterile cell scraper (Sarstedt, cat. number 83.1832) in 1x RIPA buffer (Abcam, ab156034) containing 1X HALT Protease and 1X HALT Phosphatase Inhibitor Cocktail (Thermo Scientific, cat. number 1862209, cat. number 1862495). The crude lysate was centrifuged at 14,000 rpm at 4°C for 10 minutes. The supernatant containing the total cellular protein was quantitated with Pierce BCA Protein Assay Kit (cat. number 23225) using the Infinite M200Pro multimode microplate reader (Tecan). The quantitated protein was denatured by heating in NuPAGE LDS sample buffer (Life Technologies, NP0007) for 10 min at 70°C and 40 *μ*g aliquots of the protein in sample buffer were frozen at −80°C until use.

### 2.4. SDS-PAGE and Western Blotting

20–35 *μ*g of protein was loaded along with the Precision Plus Protein Dual Colour Standards (Bio-Rad, cat. number 1610374), on Express Plus 4–12% PAGE gels (GenScript, cat. number M41210 or M41215) and electrophoresed in 1X MOPS buffer (GenScript, M00138) at 100 V. Electroblotting was performed in 1x Towbin's buffer at a constant voltage of 100 V for 1 hour at 4°C. After transfer, the PVDF membrane (Thermo Scientific, cat. number 88518) was blocked with 5% Blotto, nonfat dry milk (Santa Cruz Biotechnology, sc-2324) for 1 h at room temperature. The membrane was washed several times in PBST (1x Phosphate-buffered saline, containing 0.05% Tween 20) and incubated overnight at 4°C in appropriate primary antibodies. The membranes were probed with peroxidase conjugated with appropriate secondary antibodies. The information on antibodies is listed below. For visualization of the bands, the membranes were incubated with SuperSignal West Pico and Pierce West Femto Maximum Sensitivity chemiluminescent substrates (Thermo Scientific, cat. number 34080 and 34096) or Pierce ECL2 Western Blotting Substrate (Thermo Scientific, cat. number 80196) as per the manufacturer's instructions. The membranes were superimposed onto UltraCruz autoradiography films (Santa Cruz, cat. number sc-201697), and the films were developed using Carestream GBX developer (cat. number 1900984) and fixer (cat. number 1902485) solutions purchased from Sigma-Aldrich.

### 2.5. Antibodies

The following antibodies were used for Western blot and immunocytochemistry: Anti-Oct4 (Abcam, ab19857), anti-SOX17 antibody [3B10] (Abcam, ab 60721), anti-FOXA2 antibody (Abcam, ab 84990), anti-HNF-4-alpha antibody [K9218] (Abcam, ab 41898), anti-PARP antibody (Cell Signaling Technology, 9542), anti-caspase-3 antibody (Cell Signaling Technology, 9662), Nanog (1E6C4) Mouse mAb (Cell Signaling Technology, 4893), Sox2 (L73B4) Mouse mAb (Cell Signaling Technology, 4195), GAPDH (14C10) Rabbit mAb (Cell Signaling Technology, 2118), and anti-PAX-6 (Biolegend, 901301).

### 2.6. Immunocytochemistry

Treated cells, plated on 1.9 cm^2^ of 4-well plate (Nunc-USA), were washed twice with PBS and fixed with 3.7% formaldehyde for 12 min at room temperature. The cells were then washed twice with PBS, and permeabilized by incubating for 10 min at room temperature with PBS-2% triton-X and then incubated in blocking buffer (1% bovine serum albumin in PBS) for 15 min at room temperature. Fixed cells were then incubated with appropriate primary antibodies overnight at 4°C and then washed three times with PBS. Alexa Fluor-conjugated appropriate secondary antibodies were added to the cells for 1 h at room temperature, and after three washes with PBS, the cells were counterstained with nuclear stain DAPI (Thermo Fisher Scientific DAPI (4′,6-Diadino-2-Phenylindole, Dihhydrochloride), cat. number D1306). Fluorescent images were taken with an inverted Axiovert 40 CFL fluorescence microscope (Carl Zeiss), equipped with AxioCam HRc (Carl Zeiss).

### 2.7. Cell Viability (MTT and LDH) Assay

For cell viability assay, hESCs were cultured in 96-well tissue culture dishes coated with Matrigel. A total of 10 *μ*l of MTT (3-(4,5-dimethylthiazol-2-yl)-2,5-diphenyltetrazolium bromide) (Sigma-Aldrich, USA) (5.5 mg/ml) in PBS was dispensed into each well, and the plate was incubated at 37°C for 4.5 h. The MTT-containing medium was carefully removed, and 100 *μ*l of lysis buffer (15% SDS, 50% N,Ndimethylformamide, pH 4.7) was added to each well, incubated overnight at 37°C before the absorbance values at 590 nm were determined by a microplate reader (Perkin Elmer). Lactate Dehydrogenase (LDH) assay was performed using CytoTox 96® Non-Radioactive Cytotoxicity Assay Kit from Promega (cat. number G1780). 45 minutes prior to performing the assay, 10 *μ*l per ml of 10X Lysis solution was added to nontreated control cells and incubated at 37°C to measure the maximum amount of LDH released from the cells. The lysed cells were taken as positive control for the assay. 50 *μ*l aliquots of media from all the controls and tests were transferred in triplicates to a fresh 96-well plate. 50 *μ*l of CytoTox 96 Reagent was added to each well and incubated at room temperature for 30 minutes, protected from light. 50 *μ*l of Stop Solution was added to each well and the absorbance was measured at 490 nm, within one hour, using TECAN Infinite M200 pro plate reader.

### 2.8. TUNEL Assay

Terminal deoxynucleotidyl transferase-mediated deoxyuridine triphosphate nick end labeling (TUNEL) assay was performed on cells maintained on 96 well plates, using TACS® 2TdT-DAB In Situ Apoptosis Detection Kit from Trevigen (cat. number 4810-30-K). For hepatic differentiation, approximately 12,000 cells were seeded per well; whereas for neural differentiation, cells were seeded to attain 100% confluency within 24 hours postseeding. Cells were rinsed with 1x PBS and fixed with 3.7% buffered formaldehyde solution at room temperature for 10 minutes. Cells were washed with 1x PBS and permeabilised with 50 *μ*l Proteinase K solution (diluted 1 : 50 with deionized water), for 15 minutes at room temperature. The cells were washed twice with deionized water, and endogenous peroxidase was quenched by incubating in quenching solution of 30% hydrogen peroxide (Sigma-Aldrich, H1009) in methanol for 5 minutes at room temperature. After rinsing the cells with 1x PBS for 1 minute, cells were equilibrated in 1x TdT Labeling buffer for 5 minutes at room temperature. The cells were labelled using appropriate TdT Labeling Reaction Mix at 37°C for 1 hour in a humidity chamber. For the TUNEL negative control, nontreated cells were labeled with the Labeling Master mix devoid of the Terminal deoxynucleotidyl transferase enzyme. Antibiotics-treated cells were labeled with labeling reaction mix comprised of dNTPs, 50X manganese cation, and terminal deoxynucleotidyl transferase enzyme in 1x TdT Labeling buffer. As a TUNEL positive control, nonantibiotic-treated cells were labeled with the labeling reaction mix containing nuclease enzyme. Labeling reaction was stopped by incubating cells in 1x TdT Stop buffer for 5 minutes at room temperature. The cells were washed twice in deionized water for 5 minutes and incubated with 50 *μ*l of Strep-HRP antibody (diluted in 1x PBS) for 30 minutes at room temperature. The cells were washed twice in 1x PBS for 2 minutes each and incubated in DAB solution at room temperature for 7 minutes. The cells were washed several times with deionized water, and images were captured using 20x phase objective of EVOS XL Core Cell Imaging System (Thermo Fisher Scientific).

### 2.9. Flow Cytometric Analysis

H9 cells were cultured in feeder-free condition on Matrigel-coated 24-well tissue culture plates (Corning, cat. number 3527) in mTesR1 media for 5 days. Cells were stained with anti-Oct4 primary antibody (Abcam, cat. number ab19857) and visualized with secondary IgG (Goat anti-Rabbit IgG (H+L), Alexa Fluor 488 conjugate, Millipore (cat. number AP132J44). The flow cytometric data for the stained cells was acquired on FACSCanto II flow cytometers (BD Biosciences, USA). Data collected on 10,000 cells were analyzed using FACSDiva software (BD Biosciences).

### 2.10. Statistical Analysis

All experiments were performed in triplicates. Results shown are mean ± standard deviation (SD). Statistical analyses were made using GraphPad software (two-tailed unpaired Student's *t*-test) with asterisks representing differences being significant (^∗^*p* ≤ 0.05, ^∗∗^*p* ≤ 0.01, and ^∗∗∗^*p* ≤ 0.001).

## 3. Results

### 3.1. Effect of Gentamicin and Penicillin-Streptomycin on hESC Proliferation

**Figure 1:**
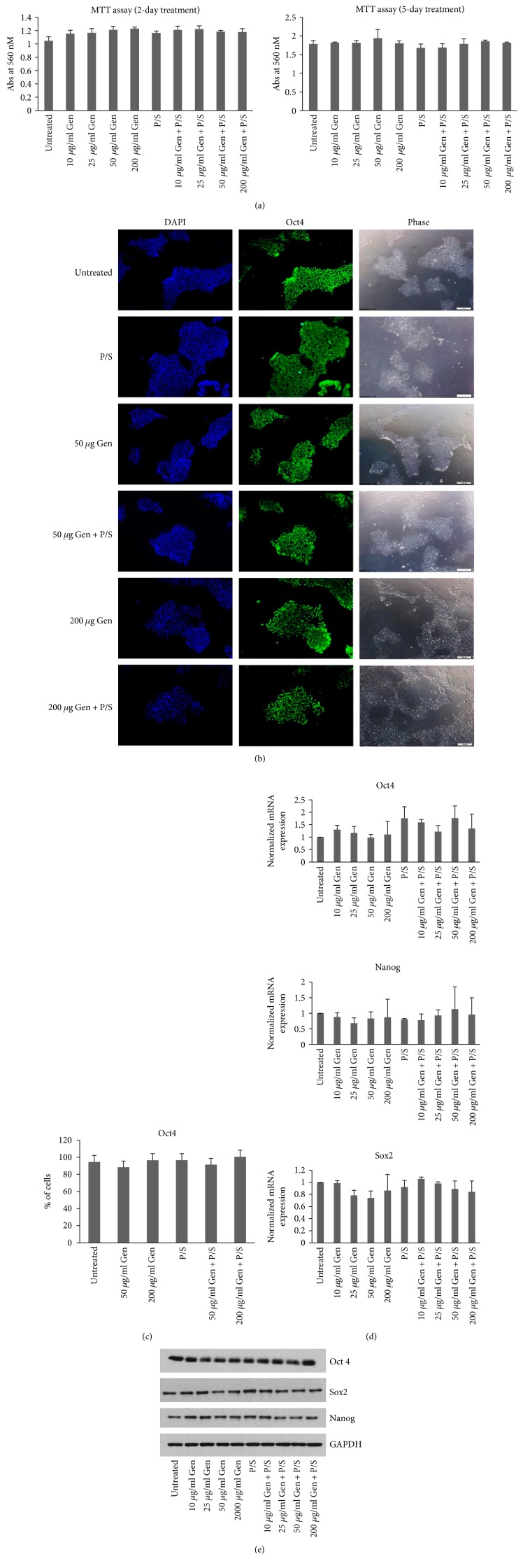
Treatment of H9 cells with antibiotics does not affect their viability, morphology, and the expression of pluripotency markers. (a) MTT assay was performed on H9 cells (passage number 40) grown in 96 well plates, at day 2 or day 5, posttreatments. Bars represent absorbance recorded at 560 nM. (b) Cells (passage number 40) were grown in feeder-free condition on Matrigel-coated plates for 5 days treated with antibiotics as indicated. The cells were fixed and photographed using inverted microscope in the bright field to compare colony morphology. The cells were then stained using anti-Oct4 antibody and imaged with fluorescence microscope. DAPI represents nuclear staining. Scale bar = 100 *μ*m. (c) The Oct4-stained cells were quantified using FACS. The bars represent percentage of cells positive for Oct4 in comparison to no antibody control. (d) The expression of RNA on cells (passage number 40) treated with antibiotic concentrations as indicated on day 5 was analyzed by qRT-PCR using primers specific for Oct4, Sox2, and Nanog genes. The expression of Polr2g gene was used as normalizer. The bars represent normalized mRNA expression with values of untreated samples set as 1. (e) The protein levels of Oct4, Sox2, and Nanog were analyzed as indicated. The expression of GAPDH was used as loading control. Each experiment was performed at least three times. Values are expressed as mean ± SD in each group.

In order to understand the effect of the antibiotics, gentamicin and pen-strep on the growth and viability of hESCs, the most widely used hESC line, H9 cells were grown in feeder-free conditions on mTeSR1 medium and treated with different concentrations of gentamicin ranging from 0, 10, 25, 50, and 200 *μ*g/ml combined without or with pen-strep (100 U/ml-100 *μ*g/ml) for 2 and 5 days to test the effect on cell viability. MTT assay was performed in a 96 well plate by seeding 20,000 cells per well in triplicate for each antibiotic concentration. No difference in cell viability was observed with the treatment of different concentrations of gentamicin alone or in combination with pen-strep including those treated with a high concentration of gentamicin, that is, 200 *μ*g/ml alone or combined with pen-strep (Figure 1(a)). In order to identify the effect of these antibiotics on pluripotency or the colony formation of hESCs, the cells were seeded in 1.9 cm^2^ well tissue culture dishes and treated with antibiotic concentrations as stated above for 5 days. Phase-contrast images were captured to visualize the colonies. The cells were then fixed and stained using antibody for pluripotency marker Oct4 (Figures 1(b) and 1(c)). No difference in the colony formation efficiency or the levels of Oct4 expression as quantitated by FACS was observed in any of the treated wells. Further, changes if any in the expression of pluripotency markers, Oct4, Sox2, and Nanog were analyzed at the RNA level by qRT-PCR and protein level by Western blotting. No significant difference in the expression of any of these markers was observed either at RNA or protein levels in the antibiotic-treated samples when compared to the untreated control (*p* values > 0.05) (Figures 1(d) and 1(e)).

Together, these results suggest that the treatment of hESCs with the antibiotics gentamicin or pen-strep has no effect on their viability, pluripotency, or the colony forming abilities.

### 3.2. Effect of Gentamicin and Penicillin-Streptomycin on Neural Fate Specification

**Figure 2:**
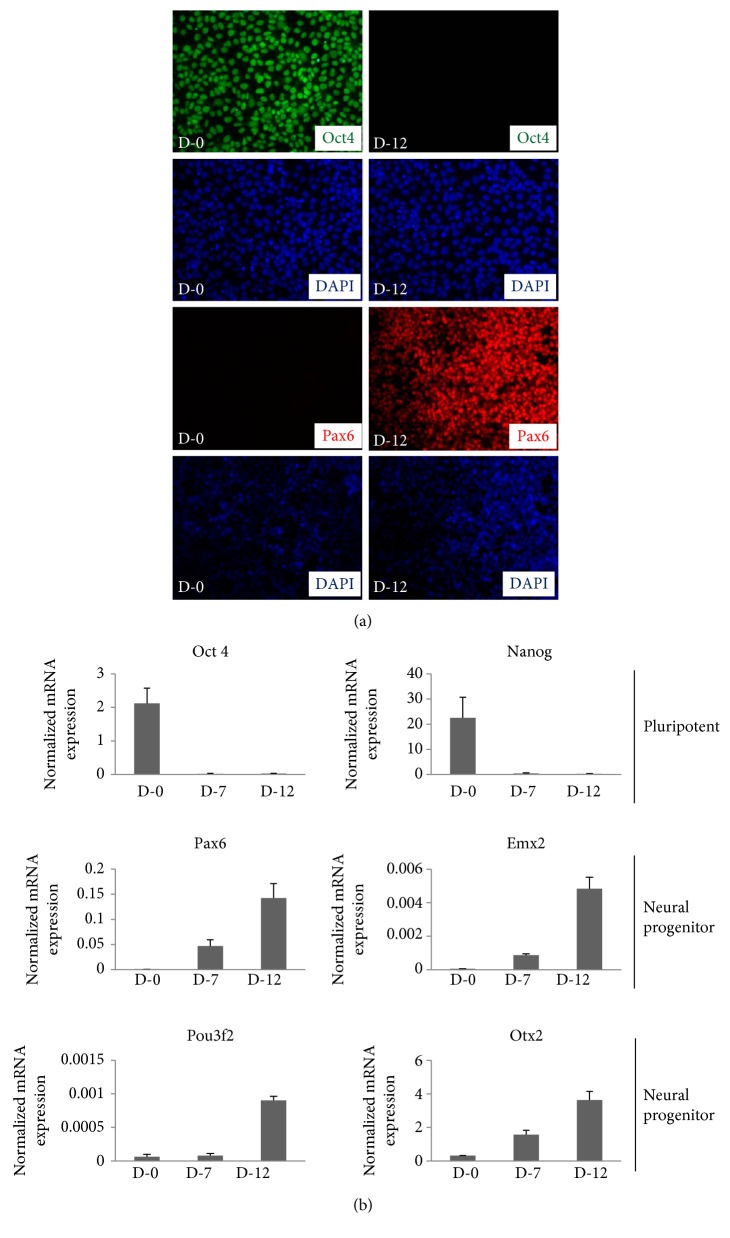
Establishment of directed differentiation of H9 cells towards neural fate using dual SMAD inhibition protocol. (a) Cells were collected from day 0 (passage number 37) and day 12 (passage number 39) of differentiation for immunostaining using antibodies against Oct4 and Pax6. The cells were imaged with fluorescence microscope. DAPI represents nuclear staining. Scale bar = 100 *μ*m. (b) Cells were collected for RNA isolation from days 0, 7, and 12 of differentiation. The mRNA expression of pluripotency markers, Oct4 and Nanog, and neural stem cell-specific markers Pax6, Emx2, Pou3f2, and Otx2 were analyzed by qRT-PCR using gene-specific primers. The bars represent fold expression of indicated genes over normalizing control, Polr2g. Values are expressed as mean ± SD in each group. The results are representative of at least three independent differentiation experiments.

**Figure 3:**
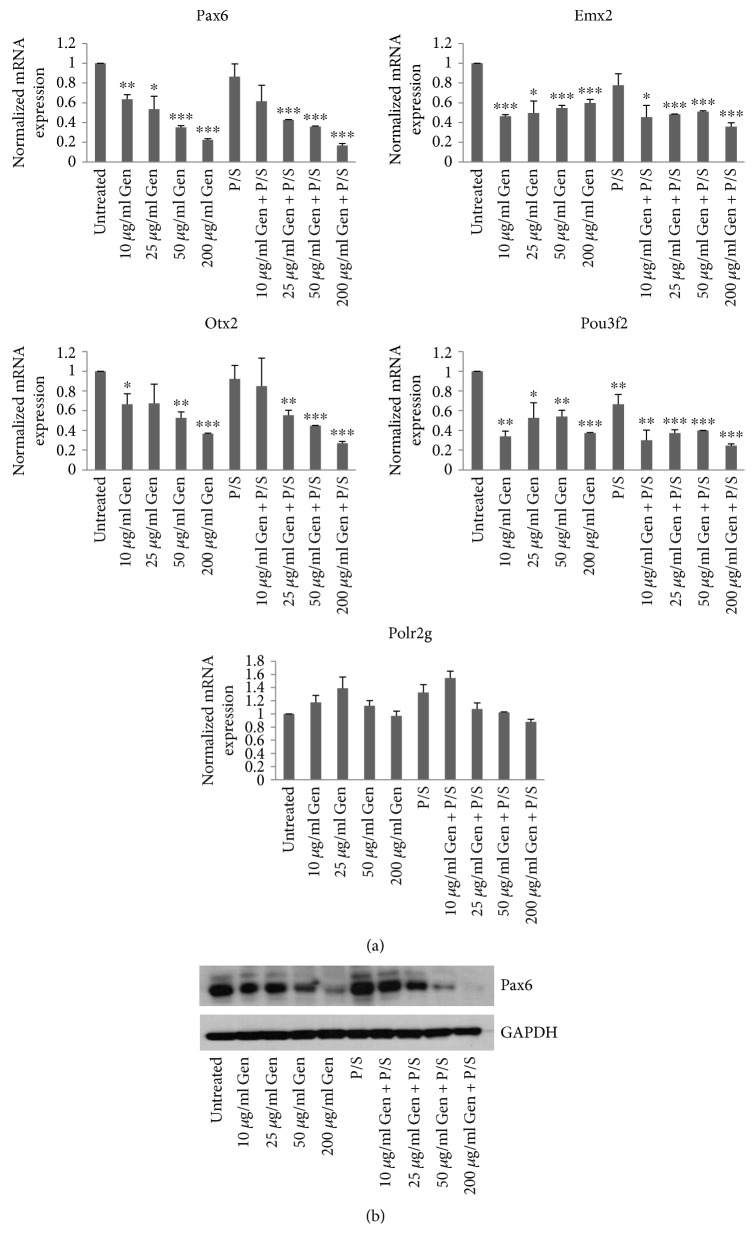
Treatment with antibiotics affects the expression of neural stem cell markers. (a) Cells (passage number 43) were collected for RNA isolation on day 10 of differentiation in the presence of antibiotics as indicated and qRT-PCR was performed to analyze the expression of Pax6, Emx2, Otx2, and Pou3f2 using gene-specific primers. The expression of Polr2g gene was used to normalize the expression. The bars represent normalized fold mRNA expression with values of untreated samples set as 1. The expression of Polr2g gene was normalized to the expression of beta-actin gene. Error bars represent standard deviation. ^∗^*p* < 0.05, ^∗∗^*p* < 0.01, and ^∗∗∗^*p* < 0.001. (b) The protein levels of Pax6 was analyzed using Western blot for samples treated with antibiotic concentrations as indicated. The expression of GAPDH was used as loading control. The results are representative of at least three independent differentiation experiments.

The neurotoxic effect of antibiotics has been reported in several previous studies, including the possibility that the use of antibiotics by the mother during pregnancy could affect the development of the central nervous system by impacting early embryonic neurogenesis (for a review, see [14]). In an effort to verify this, we replicated the neural differentiation protocol as previously used by us and others [11, 12, 15, 16]. Briefly, the H9 cells were grown on feeder-free conditions on Matrigel-coated tissue culture dishes in mTeSR1 media until the cells were fully confluent. Once confluent, neural differentiation was induced by switching mTeSR1 media to neural induction media using dual SMAD inhibition protocol which leads to the differentiation of neural stem cells of cortical fate where the expression of neural stem cell-specific markers, Pax6, Emx2, Pou3F2, and Otx2, peaks on days10–12 of differentiation [11]. Immunostaining and qRT-PCR analysis showed downregulation of pluripotency markers, Oct4 and Nanog, and upregulation of neural stem cell markers Pax6, Emx2, Pou3F2, and Otx2 on day7 and day12 of neural induction confirming that indeed the cells have differentiated towards neural fate (Figures 2(a) and 2(b)). Having confirmed the differentiation protocol, we used the same method to differentiate H9 cells in the presence of antibiotic concentrations as discussed above and reserved the cells for analysis on D-10. Total RNA was isolated and qRT-PCR was performed to check the expression of neural stem cell markers. We found that the expression of Pax6, Emx2, Pou3f2, and Otx2 genes which are known to be required for the proper neurodevelopment [12, 17] were differentially affected by different antibiotic concentrations (Figure 3(a)). The expression of Pax6 decreased with increasing concentration of gentamicin where 50 *μ*g/ml and 200 *μ*g/ml gentamicin showed the highest decrease in Pax6 expression. The treatment with pen-strep alone however did not affect the expression of Pax6, whereas the combination of pen-strep with different concentrations of gentamicin showed a similar decrease in the expression of Pax6 as seen with gentamicin alone suggesting that the decrease in the expression of Pax6 observed was mainly due to gentamicin. A similar decrease in the expression of other neural stem cell markers, Emx2, Pou3f2, and Otx2, was also observed. In an effort to test the specificity of the changes seen in the expression of these genes, we checked the expression of Polr2g gene which encodes for Rpb7 subunit of RNA Pol II and is ubiquitously expressed throughout the course of neural differentiation [12]. We did not see any significant changes in the expression of Polr2g gene in the antibiotic treated samples when compared to the untreated control suggesting that the expression changes seen for these genes due to antibiotic treatment were gene specific. Furthermore, the expression of Pax6 analyzed at protein level by Western blotting showed similar decrease in antibiotic-treated samples as seen at RNA levels by qRT-PCR (Figure 3(b)).

These results suggest that gentamicin specifically and adversely affects the expression of early neural stem cell markers and therefore its use may be detrimental to in vitro neural differentiation process.

### 3.3. Effect of Gentamicin and Penicillin-Streptomycin on Hepatic Fate Specification

**Figure 4:**
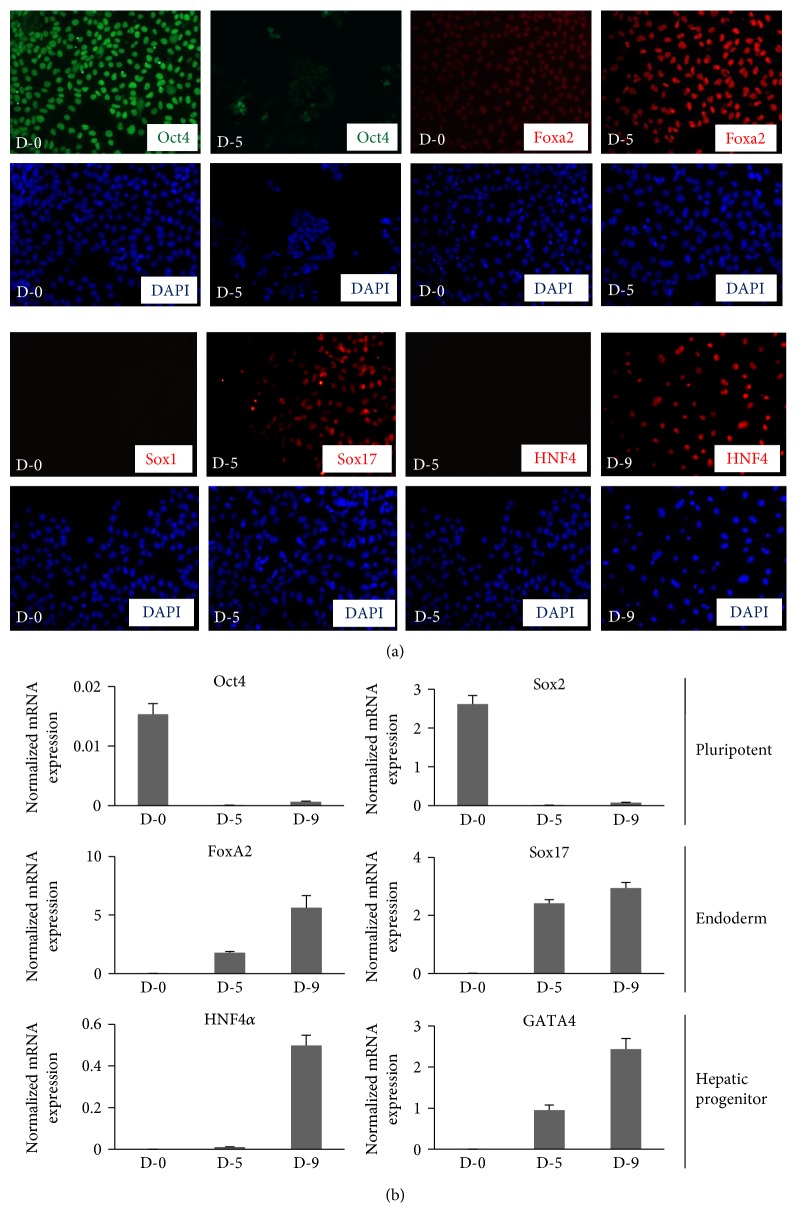
Establishment of directed differentiation of H9 cells towards hepatic fate. H9 cells (passage number 37) were differentiated towards hepatic fate using published protocols in feeder free condition on Matrigel-coated dishes. (a) Cells were collected from day 0, day 5, and day 9 of differentiation for immunostaining using antibodies against pluripotency marker Oct4; endoderm specific markers, Foxa2 and Sox17; and hepatic progenitor marker, HNF4*α*. The cells were imaged with fluorescence microscope. DAPI represents nuclear staining. Scale bar = 100 *μ*m. (b) The mRNA expression of pluripotency markers, Oct4 and Sox2; and definitive endoderm-specific markers, Foxa2 and Sox17; and hepatic progenitor markers, HNF4*α*; and GATA4 were analyzed by qRT-PCR using gene specific primers. The bars represent fold expression of indicated genes over normalizing control, Polr2g. Error bars represent standard deviation. The results are representative of at least three independent differentiation experiments.

Several protocols have been developed lately to differentiate hESCs into hepatocyte-like cells which could be useful in studies such as drug development and drug toxicity testing [18, 19]. The major steps for hESC differentiation into hepatocytes is to induce the cells for definitive endoderm (DE) followed by differentiation to hepatic progenitor cells and then into mature hepatocytes which takes about 18–25 days to complete and therefore needs addition of antibiotics in the culture media. Therefore, as tested for neural differentiation, we asked whether the presence of antibiotics would affect the expression of endoderm or hepatic markers which might eventually affect their fate. Studies in the past on animal models have shown the presence of gentamicin in fetal liver when mothers were treated with therapeutic doses of gentamicin during pregnancy [20]. Therefore, any effect of antibiotics noted on hepatogenesis in our in vitro model would also have implications on its effect in utero. We adapted the established protocols [10, 13] and grew H9 cells in feeder-free conditions to differentiate into hepatocytes. The cells were collected on day 5 to check the expression of endoderm-specific markers Foxa2 and Sox17. As shown in Figure 4(a), the expression of Oct4 has reduced significantly on day 5 of differentiation, whereas the expression of Sox17 and Foxa2 was significantly upregulated on day 5 as compared to day 0. Media was then switched to hepatocyte progenitor media for 5 more days. After which the cells were collected and checked for the expression of hepatic progenitor marker, HNF4*α,* which was found to be significantly up regulated on day 9 as compared to day 5 (Figure 4(a)). These markers of differentiation were further confirmed by qRT-PCR (Figure 4(b)). Together, these results confirmed the feasibility of hepatic differentiation protocol.

**Figure 5:**
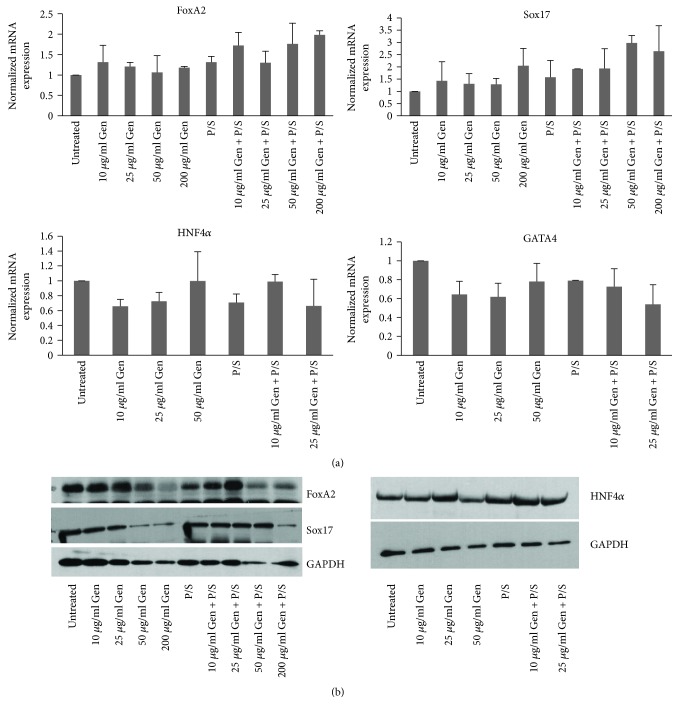
Effect of antibiotic treatment on the expression of markers of definitive endoderm and hepatic progenitor fate. H9 cells (passage number 39) were differentiated towards definitive endoderm and hepatic fate in the presence of antibiotics throughout the course of differentiation as indicated. (a) Cells were collected for RNA isolation on day 5 (endoderm) and day 9 (hepatic progenitors) of differentiation in the presence of antibiotics and qRT-PCR was performed to analyze the expression of endoderm specific markers, Foxa2 and Sox17, and hepatic progenitor markers, HNF4*α* and GATA4, using gene specific primers. The expression of Polr2g gene was used as normalizer. The bars represent normalized fold mRNA expression with values of untreated samples set as 1. The results are representative of three independent differentiation experiments. (b) The protein expression levels of Foxa2 and Sox17 (samples from day 5) and HNF4*α* (samples from day 9) were analyzed using Western blot for samples treated with antibiotic concentrations as above. The expression of GAPDH was used as loading control. The results are representative of three independent experiments.

Using this method, we treated H9 cells with different antibiotic concentrations of gentamicin with or without pen-strep as before and differentiated them towards hepatic fate. Total RNA was isolated from day 5 and day 9 of differentiation, and qRT-PCR was performed for Foxa2 and Sox17 from day 5 samples. No significant difference (*p* > 0.05) in the RNA expression was observed for these markers from samples treated at 10 and 25 *μ*g/ml gentamicin combined with or without pen-strep as compared to untreated samples. However, cells treated with 50 and 200 *μ*g/ml gentamicin combined with pen-strep showed around 2.0 to 2.9-fold increase in expression for both genes compared to untreated samples. However, these differences were not consistent (Figure 5(a)). We therefore concluded, although increased expression of Foxa2 and Sox17 was observed with high doses of gentamicin plus pen-strep treatment, these differences were not significant. The RNA collected on day 9 of differentiation was checked for hepatic progenitor markers HNF4*α* and GATA4. Importantly, the cells after treatment with antibiotics showed significant levels of cell death upon hepatic differentiation. The cell death at antibiotic concentrations, 200 *μ*g/ml gentamicin and 50 and 200 *μ*g/ml gentamicin combined with pen-strep, was so high that there were not many cells left from day 9 of differentiation for further analysis. The RNA expression levels for HNF4*α* and GATA4 however did not show any marked differences in the expression at other antibiotic concentrations when compared with untreated samples (Figure 5(a)). Furthermore, expression analysis of Sox17, Foxa2, and HNF4*α* at protein level by Western blotting from samples at day 5 and day 9 of differentiation showed similar results as observed by qRT-PCR (Figure 5(b)). These results suggest that antibiotics gentamicin and pen-strep do not affect the expression of endoderm and hepatic progenitor markers during the course of in vitro hepatogenesis.

### 3.4. Gentamicin Causes Cellular Apoptosis during In Vitro Cell Differentiation

**Figure 6:**
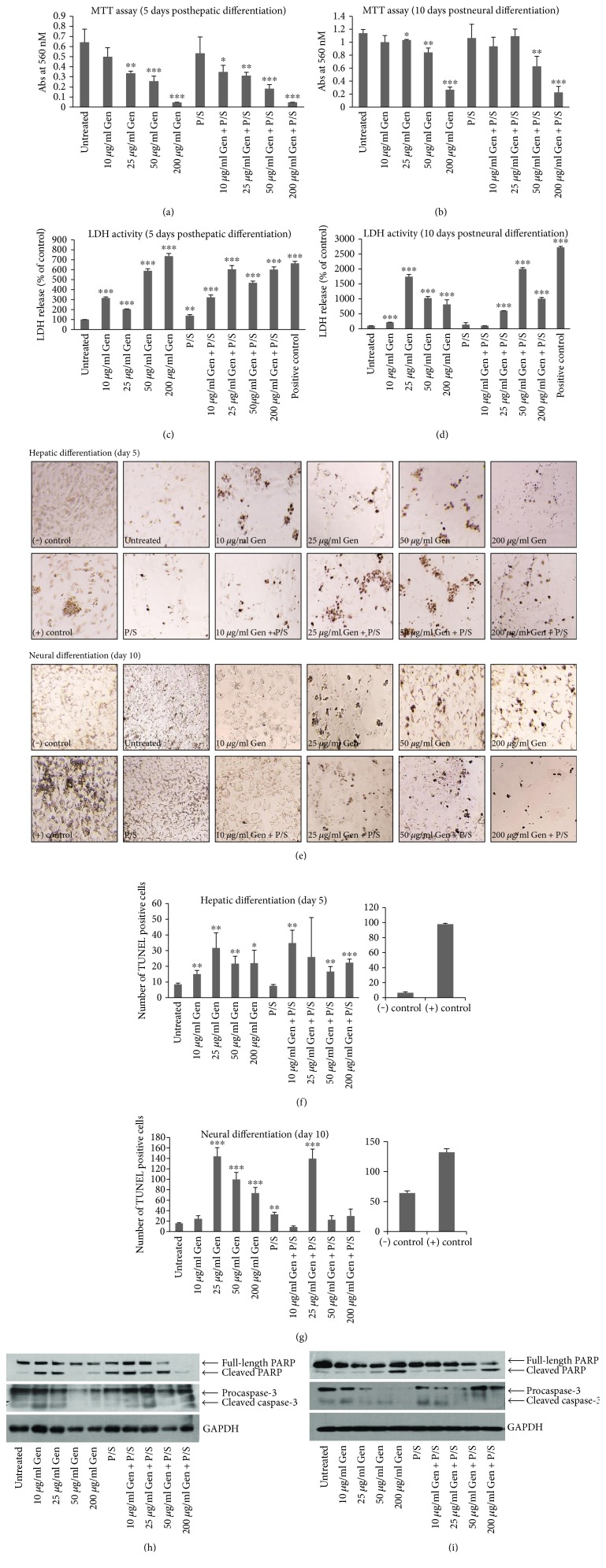
Antibiotic treatment causes cellular apoptosis during differentiation of H9 cells towards hepatic and neural fate. (a), (b) The cells (passage number 37) were seeded in 96 well plates for MTT assay upon hepatic and neural differentiation in the presence of 0, 10, 25, 50, and 200 *μ*g/ml gentamicin combined without or with pen-strep (100 U/ml-100 *μ*g/ml). The absorbance was recorded at 560 nM on day 5 and day 10 of hepatic and neural differentiation, respectively. Bars represent absorbance recorded at 560 nm. (c), (d) The release of LDH in the culture media was tested on 50 *μ*l of media from day 5 and day 10 of hepatic and neural differentiation, respectively. As positive control, nontreated cells from the same stage of differentiation were completely lysed to detect maximum LDH release. Bars represent absorbance recorded at 490 nm which is shown here as percentage of the values for untreated (without antibiotic) samples set as 100%. Error bars represent standard deviation. The results are representative of three independent experiments. ^∗^*p* < 0.05, ^∗∗^*p* < 0.01, and ^∗∗∗^*p* < 0.001. (e) For TUNEL assay, cells (passage number 44) were seeded in 96 well plates for hepatic and neural differentiation in the presence of 0, 10, 25, 50, and 200 *μ*g/ml gentamicin combined without or with pen-strep (100 U/ml-100 ug/ml). Images for TUNEL positive cells were captured by light microscope. (f), (g) Quantitation for TUNEL assay was made by counting TUNEL positive cells from each antibiotic treated well (five fields, one in the center and four in each corners of the well). (h), (i) The protein expression levels of cellular apoptotic markers PARP (full length and cleaved) and caspase-3 (pro and cleaved) were analyzed using Western blot for samples treated with antibiotic concentrations as indicated on day 5 and day 10 of hepatic- and neural-differentiated cells, respectively. The expression of GAPDH was used as loading control. The results are representative of three independent experiments. ^∗^*p* < 0.05, ^∗∗^*p* < 0.01, and ^∗∗∗^*p* < 0.001.

Aminoglycosides, gentamicin, and streptomycin are shown to cause cellular apoptosis in both animal and cell culture models mainly in the kidney tubules and sensory cells of the inner ear [2, 21–23]. As shown above, we did not observe any significant cell death during proliferation of H9 cells in mTeSR1 media supplemented with different antibiotic concentrations for five days. However, during differentiation of H9 cells towards hepatic and neural fate, we did observe significant levels of cell death especially at higher concentrations of gentamicin and gentamicin combined with pen-strep (Figure S1 available online at https://doi.org/10.1155/2017/2451927). Especially during hepatic differentiation procedure, the cell death was so severe that we were unable to collect cells at day 9 of differentiation from samples treated with 50 and 200 *μ*g/ml gentamicin alone or combined with pen-strep for further analysis. Therefore, to identify the effect of these antibiotics on cell death during differentiation, we cultured and differentiated H9 cells towards neural and hepatic fate using the same protocol as discussed above in 96-well plates and assayed them for cell viability. Cells differentiating toward hepatic fate were collected on day 5, and those towards neural fate were collected on day 10 of differentiation for MTT assay. The results of MTT assay showed reduced cell viability at 25, 50, and 200 *μ*g/ml gentamicin as well as gentamicin and pen-strep combined. The cell viability decreased steadily with an increase in gentamicin concentration from 25 to 200 *μ*g/ml for both hepatic and neural differentiation of H9 cells. The lower concentration of gentamicin (10 *μ*g/ml) alone or combined with pen-strep or pen-strep alone did not cause significant reduction in cell viability when compared with untreated controls (Figures 6(a) and 6(b)). Further, to confirm whether reduced cell viability due to antibiotic treatment results from increased cell death, we performed LDH assay. The amount of LDH released in the culture media due to a damaged cell membrane is indicative of the amount of cell death. We found that treatment with antibiotics causes significantly increased amounts of LDH release both during hepatic as well as neural differentiation (Figures 6(c) and 6(d)). Even the lowest concentration of gentamicin used in this study (10 *μ*g/ml) showed significantly elevated levels of LDH release during differentiation. The antibiotic-induced cell death during hepatic and neural differentiation was also assessed by TUNEL assay which is used to detect damaged DNA strands usually associated with apoptotic cell death. We found increased number of TUNEL positive cells starting from 10 *μ*g/ml gentamicin alone or combined with pen-strep during hepatic differentiation; however, for neural differentiation, the increase was observed from 25 *μ*g/ml gentamicin alone or combined with pen-strep (Figures 6(e), 6(f), and 6(g)) compared with untreated control. These results suggest that the cause of death due to antibiotics could be apoptic in nature. Previous studies have shown that gentamicin and other aminoglycosides cause cellular apoptosis by the activation of caspase cascade [2, 24]. To test whether activation of caspases is responsible for cell death observed, we analyzed the expression of well-known apoptotic markers pro- and cleaved caspase-3 by Western blotting using cell lysates collected from both hepatic and neural differentiated cells treated with different antibiotic concentrations as stated above. We also checked by Western blotting the expression of full length and cleaved Poly ADP-ribose polymerase (PARP) on the same lysate and compared it with GAPDH as control to confirm cellular apoptosis [25–27]. A steady decrease in the levels of procaspase-3 and an increase in the levels of active caspase-3 expression upon treatment with the antibiotics gentamicin or gentamicin combined with pen-strep were observed during both neural and hepatic differentiation (Figures 6(h) and 6(i)). The levels of active PARP also increased similarly after treatment with antibiotics compared to the untreated control. Interestingly, although low concentrations of gentamicin (10 *μ*g/ml alone or combined with pen-strep) did not show significant levels of TUNEL positive cells during neural differentiation, there were increased levels of active PARP and cleaved caspase-3 at this antibiotic concentration both in hepatic- and neural-differentiated cells as compared to untreated cells. This suggests that an even a low concentration of gentamicin is able to induce cellular apoptosis upon differentiation of H9 cells. Overall, these results suggest that the treatment of H9 cells with gentamicin or combined with pen-strep causes significant cellular apoptosis by the induction of caspase-3 upon differentiation towards hepatic as well as neural fate.

## 4. Discussion

Although, a host of studies have tested the effect of aminoglycoside antibiotics on in vitro culture and differentiation [28–31] of stem cells such as bone marrow- or adipose tissue-derived stem cells as well as murine ESCs, as per our knowledge no study has been conducted so far to test the effect of aminoglycoside antibiotics on hESC proliferation and differentiation. A common outcome of these earlier studies was cytotoxicity and defective lineage specific differentiation, two important observations noted in our study on hESCs as well. Our results suggest that treatment of H9 cells with antibiotics does not show any effect on cell viability or expression of pluripotency markers, Oct4, Sox2, and Nanog, both at RNA and protein levels. In contrary to this, directed differentiation of these cells towards neural as well as hepatic fate showed significant cell death at different gentamicin concentrations which increased gradually suggesting dose dependency. The question then arises, why there is no change in the proliferation and cell viability of undifferentiated cells by the antibiotics although upon differentiation, these cells are susceptible to these antibiotics. One possibility may be that H9 cells are adapted to antibiotics in their pluripotent state. Although the WiCell Institute from where these cells were obtained does report maintaining these cells in the presence of pen-strep, there is no report of them being exposed to gentamicin. After obtaining the cells from WiCell, these cells were never cultured in any type of antibiotics in our lab except for this study. Therefore, they could not have been adapted to gentamicin. However, it may be possible that undifferentiated pluripotent cells have the potential to better adapt to teratogen exposure as compared to during their differentiation. Undifferentiated ESCs are pluripotent cells with the potential to be differentiated into all three germ layers. Therefore, cell survival mechanisms in undifferentiated ESCs might be more precise than differentiated cells to maintain genome integrity to avoid transfer of errors to differentiated progeny [32–34]. Thus, undifferentiated ESCs may have cell survival mechanisms different from their differentiated progeny [33] and may be more resistant to the effect of teratogens [35]. This view is supported by the studies conducted in the past initially on murine and later on human ESCs which showed that the cellular defense mechanisms due to various stresses are superior in undifferentiated ESCs in comparison to differentiated cells [35, 36]. It was argued that the efficient response to stress in undifferentiated cells could result from low levels of stress generation in these cells as well as highly active stress defense mechanisms [37]. Another possibility is that, accumulation of DNA damage or mutation in these cells due to teratogens may lead to their spontaneous differentiation or apoptosis to remove affected cells from stem cell population. However, upon treatment of H9 cells with antibiotics, we neither see increased cell death nor reduced levels of pluripotency markers suggesting that antibiotics do not cause spontaneous differentiation or apoptosis in these cells. Therefore, tolerance of undifferentiated ESCs to antibiotics in our experiments could be due to higher efficiency of cellular defense mechanisms in these cells in comparison to during differentiation. Indeed, previous results in both murine and human ESCs show that these cells are equipped with highly efficient antioxidant defense mechanisms to combat reactive oxygen species (ROS) production due to higher expression levels of antioxidant enzymes as well as lower numbers of mitochondria and less reliance on oxidative phosphorylation, all of which diminish significantly during differentiation [37, 38]. One of the molecular mechanisms through which aminoglycoside antibiotics induce apoptotic signaling cascade in mammalian cells is through excessive generation of ROS [39, 40]. It has been shown that gentamicin forms a complex with iron (Fe^III^) in the cytosol and coverts it to Fe^II^. This Fe^II^-gentamicin complex associates with membrane lipids such as arachidonic acid leading to its peroxidation resulting in excessive ROS generation in the presence of molecular oxygen. Therefore, increased ROS production due to antibiotics and reduced antioxidant defense mechanisms in differentiating ESCs together could have resulted in increased cell death observed in our studies upon differentiation towards both hepatic and neural lineages. Furthermore, it was also argued that the higher efficiency of stress tolerance in undifferentiated ESCs could be due to the involvement of cellular stress responsive, heat shock protein-70 (HSP70) family of proteins which express at a high level in undifferentiated ESCs but are downregulated in differentiated cells [37, 38, 41]. HSPs are a group of proteins which are induced by a variety of chemical and physiological stresses to protect the cells from damage by their ability to act as protein chaperones by preventing protein misfolding as well as their antiapoptotic function due to inhibition of the activation of caspase cascade as reported for HSP70 [42, 43]. Towards this end, a study in mice has shown that constitutive overexpression of HSP70 inhibits aminoglycoside-induced hair cell death [44]. To check whether HSP70 genes are expressed in undifferentiated ESCs and whether their levels change upon differentiation, we used genome-wide transcriptomic data published by Joyce et al. ([12], http://cortecon.neuralsci.org/) on H9 differentiation into neurons. We found that indeed the expression of HSP70-2, HSP70-4, and HSC70 (HSP73) genes are substantially downregulated upon neural differentiation in comparison to undifferentiated ESCs (Figures S2A, B, and C). Since we have used the same method of neural differentiation as used by Joyce et al., we presume that the levels of these HSP70s would have been downregulated upon neural differentiation in our experiments. Therefore, high expression levels of HSP70 proteins in undifferentiated ESCs could be one mechanism through which these cells are able to combat the apoptotic trigger induced by aminoglycoside antibiotics.

Another mechanism could be through direct interaction of aminoglycosides with chaperon proteins, HSP73, calreticulin, and CLIMP-63 [40], which results in their inactivation. The function of these chaperon proteins is to help protein folding and quality control in the endoplasmic reticulum, and a defect in this could induce unfolded protein response pathway which may result in apoptosis. It has been shown that the chaperone activity of both HSP73 and calreticulin is inhibited by gentamicin [40]. Furthermore, gentamicin causes dimerization and inactivation of CLIMP-63 in kidney tubule cells causing gentamicin-induced cytotoxicity [45]. Although the expression levels of calreticulin and CLIPM-63 did not change upon neural differentiation, the expression of HSP73 was reduced by more than twofold (Figure S2C). Therefore, combined effect of low levels of HSP73 as well as antichaperone activity of aminoglycosides may lead to aminoglycoside-mediated cytotoxicity in differentiating cells as observed in our studies. However, since the levels of calreticulin and CLIP-63 were the same in both undifferentiated and upon neural differentiation of hESCs, it is unlikely that antichaperone activity of gentamicin due to interaction with these two proteins will selectively cause cytotoxicity upon differentiation but not in undifferentiated cells.

In addition to intracellular mechanisms of aminoglycoside-mediated cytotoxicity as discussed above in undifferentiated versus differentiating ESCs, other mechanisms may be in the differences in the uptake of antibiotics in the two cell types. Gentamicin binds to specific surface proteins in mammalian cells and enters into the cytosol through endocytosis [46]. In proximal kidney tubule cells, gentamicin binds to a multiligand binding receptor, megalin or LRP2, resulting in endocytosis and renal accumulation of gentamicin [47]. Interestingly, we found that LRP2 does not express in undifferentiated H9 cells; however, its expression at RNA level is upregulated severalfold upon neural differentiation (Figure S2D) suggesting that another mechanism of cytotoxicity observed in our study could be due to the differences in the uptake of aminoglycosides which might be higher in the differentiating ESCs. However, the levels of these proteins involved in cellular response to aminoglycoside antibiotics as discussed above need to be checked during endoderm and hepatic differentiation as well to suggest whether similar regulatory mechanisms are functional during hepatic differentiation. Nonetheless, from these evidences we could still conclude that one or multiple mechanisms of aminoglycoside response might be involved resulting in cytotoxicity upon differentiation of hESCs.

The effect of antibiotics on cell viability which was observed in our studies were mediated through the activation of caspase cascade as indicated by the increase in the levels of active caspase-3 (the executioner caspase in apoptotic pathway) as well as cleaved PARP suggesting that one mechanism by which these antibiotics affect differentiating hESC is through cellular apoptosis. However, our results also suggested the possibility that the higher concentrations of these antibiotics may trigger a different cell death mechanism (50 and 200 *μ*g/ml gentamicin combined with or without pen-strep) as levels of both procaspase-3 and cleaved caspase-3 were low at these concentrations. Further, the levels of cleaved capspase-3 and cleaved PARP were higher even at lower concentrations of gentamicin with or without pen-strep where no significant cell death was observed suggesting proapoptotic conditions at these concentrations. It is of relevance to note that earlier studies testing the effect of gentamicin on kidney proximal tubular cells have reported that higher concentrations of gentamicin causes cellular necrosis while lower concentrations causes apoptotic cell death [5, 48]. The mechanisms of apoptotic cell death by aminoglycoside antibiotics include phosphorylation of c-jun in JNK pathway, release of cytochrome c from mitochondria, and activation of caspase-9 and caspase-3 [5, 21, 22], whereas necrotic cell death was reported due to the absence of TUNEL and markers of classic apoptosis; instead, the presence of necrotic markers such as endonuclease G translocation and activation of calpain, and both the synthesis and activation of cathepsin D were noticed [49]. Thus, it is possible that aminoglycoside treatment may cause multiple forms of cell death depending on the concentration and duration of treatment and may not be completely dependent on the classic apoptotic pathway [23]. Therefore, apart from cell death caused by activation of caspase-3 as observed in our study, we do not rule out the possibility of other cell death mechanisms which may be active in our experiments especially at higher concentrations of gentamicin combined with pen-strep which warrant further investigation at molecular level.

Another important observation in our study is the changes seen in the expression of neural stem cell-specific markers due to the treatment of antibiotics. It has been shown before that aminoglycoside antibiotics cause mistranslation of certain proteins in eukaryotic cells by reducing the efficiency of translation termination [50, 51]. This is done by reading through the stop codon of specific mRNAs and thereby causing defective protein translation. Therefore, it can be speculated that mistranslation of some of the proteins with important roles in neural stem cell differentiation could cause defective expression of neural stem cell-specific markers as observed in this study. The hypothesis linking the use of aminoglycoside antibiotics and susceptibility of neurodevelopmental disorders such as autism supports this view [52]. Further investigation is needed to identify the mechanism of the effect of aminoglycoside antibiotics on embryonic neurogenesis.

## 5. Conclusions

From our results, we conclude that antibiotics, gentamicin, and pen-strep combination adversely affect cell viability during differentiation of hESCs towards both hepatic and neural fate. However, no effect is seen on cell viability or expression of pluripotency markers in undifferentiated ESCs. Our studies also did not show any effect on the expression of stem cell-specific markers during their differentiation towards hepatic fate; however, interestingly the expression of neural stem cell-specific markers was significantly reduced due to the antibiotics during neural differentiation. The specific effect of these antibiotics on cell viability during differentiation as well as their effect on early embryonic neurogenesis needs further investigation at molecular level.

## Supplementary Material

Figure S1. Antibiotic treatment of H9 cells during hepatic and neural differentiation causes cell death. The cells were treated with 0, 10, 25, 50 and 200ug/ml gentamicin (images 1-5 respectively) and 0, 10, 25, 50 and 200ug/ml gentamicin combined with Pen-Strep (100U/ml-100ug/ml) images 6-10 respectively. The cells were imaged in bright-field on day-5 and day-10 of hepatic and neural differentiation respectively. Figure S2. RNA expression analysis of indicated genes from H9 cells differentiated towards neurons. Number of reads from RNA-seq data for indicated genes were retrieved from CORTECON (http://cortecon.neuralsci.org/) [13] and were plotted against days post neural induction. The data are represented as mean ± standard deviation.

## Figures and Tables

**Table 1:** 

Primer name	Primer bank ID	5′-3′ sequence
Oct4-forward	4505967a2	GGGAGATTGATAACTGGTGTGTT
Oct4-reverse	4505967a2	GTGTATATCCCAGGGTGATCCTC
Nanog-forward	153945815c1	TTTGTGGGCCTGAAGAAAACT
Nanog-reverse	153945815c1	TTTGTGGGCCTGAAGAAAACT
Sox2-forward	325651854c3	TACAGCATGTCCTACTCGCAG
Sox2-reverse	325651854c3	GAGGAAGAGGTAACCACAGGG
Polr2g-forward	219879812c1	ATCTCCCTAGAGCACGAAATCC
Polr2g-reverse	219879812c1	ACAAAGCCATACTTCCCTGTG
B-actin-forward	4501885a1	CATGTACGTTGCTATCCAGGC
B-actin-reverse	4501885a1	TCTTCATGAGGTAGTCAGTCAGGT
Pax6-forward	189083679c1	TGGGCAGGTATTACGAGACTG
Pax6-reverse	189083679c1	ACTCCCGCTTATACTGGGCTA
Emx2-forward	164607120c1	CGGCACTCAGCTACGCTAAC
Emx2-reverse	164607120c1	CAAGTCCGGGTTGGAGTAGAC
Pou3f2-forward	380254475c2	AAGCGGAAAAAGCGGACCT
Pou3f2-reverse	380254475c2	GTGTGGTGGAGTGTCCCTAC
Otx2-forward	27436932c1	CAAAGTGAGACCTGCCAAAAAGA
Otx2-reverse	27436932c1	TGGACAAGGGATCTGACAGTG
Foxa2-forward	194363755c1	GGAGCAGCTACTATGCAGAGC
Foxa2-reverse	194363755c1	CGTGTTCATGCCGTTCATCC
Sox17-forward	145275218c1	GTGGACCGCACGGAATTTG
Sox17-reverse	145275218c1	GGAGATTCACACCGGAGTCA
HNF4*α*-forward	71725338c2	CGAAGGTCAAGCTATGAGGACA
HNF4*α*-reverse	71725338c2	ATCTGCGATGCTGGCAATCT
GATA4-forward	172072611c1	CGACACCCCAATCTCGATATG
GATA4-reverse	172072611c1	GTTGCACAGATAGTGACCCGT

## Abbreviations

hESCs: Human embryonic stem cells
pen-strep: Penicillin-streptomycin.
