# Supplementary material for: Effects of Aminoglycoside Antibiotics on Human Embryonic Stem Cell Viability during Differentiation In Vitro
Source: Stem Cells Int. 2017 Sep 24;2017:2451927. doi: 10.1155/2017/2451927 (PMC5632925; doi:10.1155/2017/2451927)

Figure S1. Antibiotic treatment of H9 cells during hepatic and neural differentiation causes cell death. The cells were treated with 0, 10, 25, 50 and 200ug/ml gentamicin (images 1-5 respectively) and 0, 10, 25, 50 and 200ug/ml gentamicin combined with Pen-Strep (100U/ml-100ug/ml) images 6-10 respectively. The cells were imaged in bright-field on day-5 and day-10 of hepatic and neural differentiation respectively.

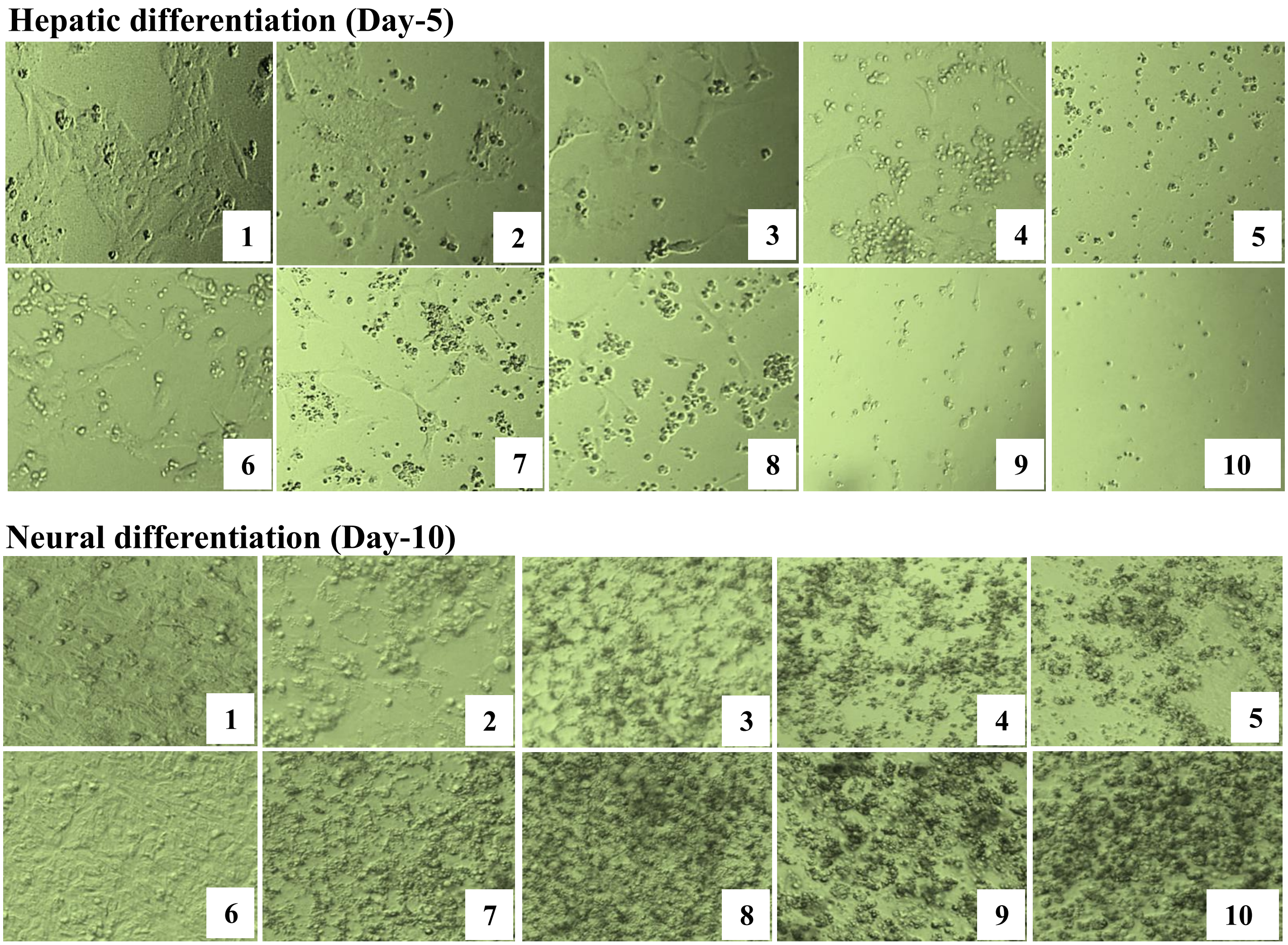

Figure S2. RNA expression analysis of indicated genes from H9 cells differentiated towards neurons. Number of reads from RNA-seq data for indicated genes were retrieved from CORTECON (http://cortecon.neuralsci.org/) [13] and were plotted against days post neural induction. The data are represented as mean ± standard deviation.

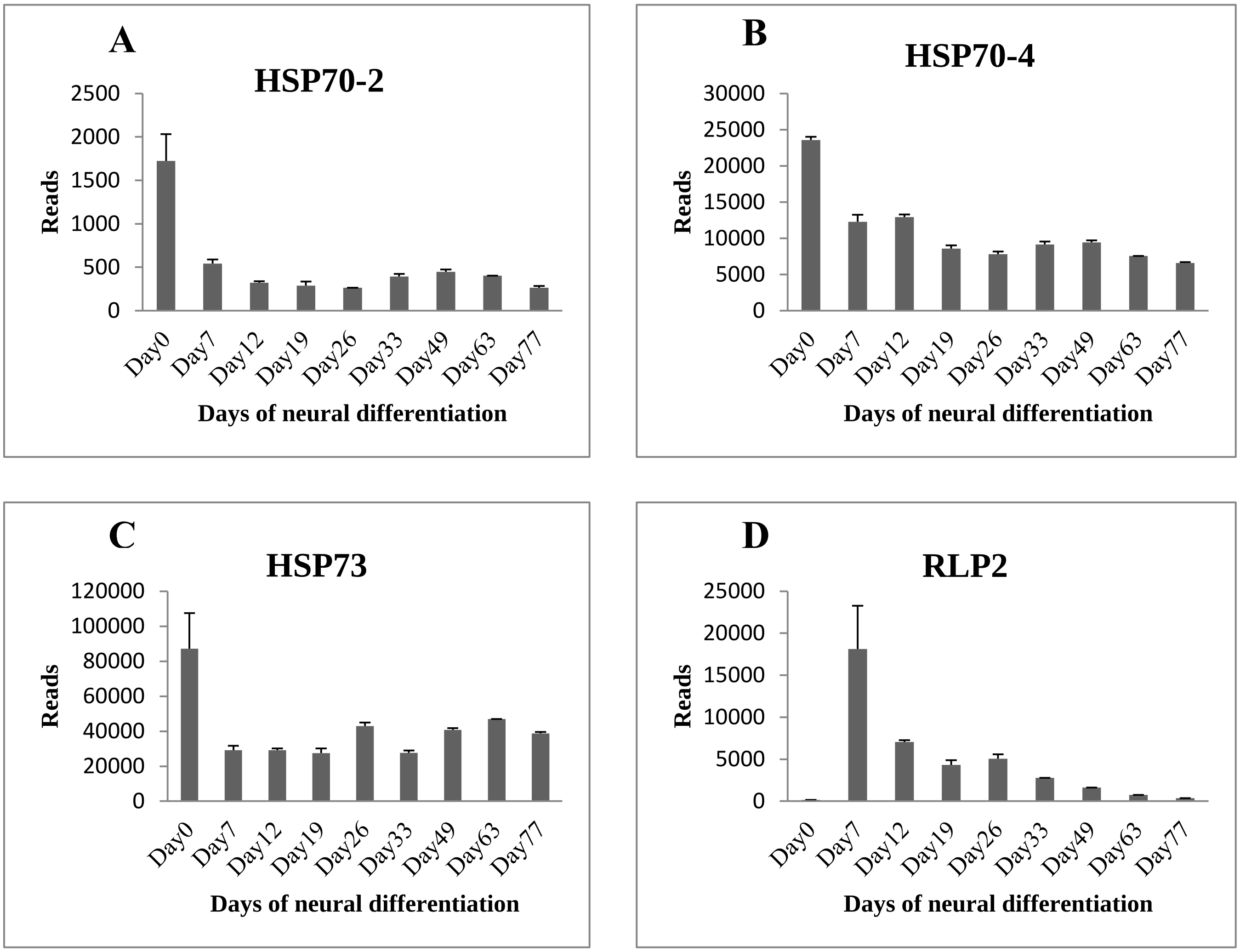

Supplement: Supplementary file 1 — Figure S1. Antibiotic treatment of H9 cells during hepatic and neural differentiation causes cell death. The cells were treated with 0, 10, 25, 50 and 200ug/ml gentamicin (images 1-5 respectively) and 0, 10, 25, 50 and 200ug/ml gentamicin combined with Pen-Strep (100U/ml-100ug/ml) images 6-10 respectively. The cells were imaged in bright-field on day-5 and day-10 of hepatic and neural differentiation respectively. Figure S2. RNA expression analysis of indicated genes from H9 cells differentiated towards neurons. Number of reads from RNA-seq data for indicated genes were retrieved from CORTECON (http://cortecon.neuralsci.org/) [13] and were plotted against days post neural induction. The data are represented as mean ± standard deviation. [file 2451927.f1.pdf]
